# Diagnosis of* Helicobacter pylori* Using Invasive and Noninvasive Approaches

**DOI:** 10.1155/2018/9064952

**Published:** 2018-05-22

**Authors:** Amin Talebi Bezmin Abadi

**Affiliations:** Department of Bacteriology, Faculty of Medical Sciences, Tarbiat Modares University, Tehran, Iran

## Abstract

*Helicobacter pylori (H. pylori)* as gram-negative and spiral microorganism is responsible for colonization in the gastric microniche for more than 50% of world population. Recent studies have shown a critical role of* H. pylori* in the development of peptic ulcers, gastric mucosa-associated lymphoid tissue (MALT) lymphoma, and gastric cancer. Over the past decade, there has been a sharp interest to use noninvasive tests in diagnosis of the* H. pylori* infection. During the years after discovery by Marshall and Warren, it has been frequently declared that the rapid urease test (RUT) is one of the cheapest and rapid diagnostic approaches used in detecting the infection. Although the specificity and sensitivity are durable for this test, clinical experiences had shown that the ideal results are only achieved only if we take biopsies from both corpus and antrum at the same time. Given the diagnosis of the* H. pylori* in clinical samples, gastroenterologists are facing a long list of various molecular and nonmolecular tests. We need more in-depth researches and investigations to correctly generalize rapid and accurate molecular tests determining both bacterial identity and antibiotic resistance profile.

## 1. Introduction

Following the groundbreaking discovery of* Helicobacter pylori (H*.* pylori)* in 1983, a challenging era in the management of gastroduodenal diseases has been initiated [1]. This usually chronic infection is thought to play an inevitable role in peptic ulcer diseases and gastric adenocarcinoma.* H. pylori,* as the most commonly prevalent and recognized bacterium, is carried by more than half of the world population [2–6]. Once colonized,* H. pylori* induces a persistent, but superficial, inflammation, resulting in duodenal ulcer, gastric ulcer, and gastric cancer [7–11]. As predicted, many recent studies have confirmed a critical role of* H. pylori* in the development of peptic ulcers, gastric mucosa-associated lymphoid tissue (MALT) lymphoma, and gastric cancer [12]. Given the causative role of* H. pylori* in duodenal ulcer and gastric cancer, clinicians and microbiologists are eager to find the best diagnostic approach [13–18]. Currently, there are various diagnostic methods used for* H. pylori* infection in different subjects (children and adults), but the only methods with both high sensitivity and high specificity remain useful and recommendable. In other words, precisive detection of this bacterium in different clinical specimens (e.g., urine, stool, saliva, biopsy, and gastric juice) attributed with successful therapeutic practice will be listed in hot topic researches interest globally [19–22]. According to a traditional classification,* H. pylori* infection can be diagnosed by noninvasive tests such as* H. pylori *antigen in stool specimen, UBT (Urea Breath Test), serology, and invasive tests such as PCR (polymerase chain reaction), culture, and histology which require endoscopic surgery and biopsy specimens [23–25]. Invasive tests (e.g., Histological examination, culture, and polymerase chain reaction) require endoscopy and noninvasive techniques (e.g., serology and urea breath) are independent of endoscopic surgery. Nonetheless, for having the best management of* H. pylori*-related diseases, we need to specific and accurate diagnosis, especially for treatment courses (pretreatment and posttreatment of* H. pylori* infection). In fact, the selection of choice method is highly dependent on the availability and feasibility of many circumstances [26]. To now, many tests had been invented for diagnosis of* H. pylori*; however, each one has certain advantages and disadvantages [26–29]. In current article, we will review the recent advances of invasive and noninvasive methods suggested in diagnosis of* H. pylori*. Moreover, application and priorities of those methods, especially in evaluating the infection following the therapeutic regimen, are secondary goals of our paper.

## 2. Endoscopy: A Pivotal Approach in Diagnosis of* H. pylori*

Although various methods had been attempted to accurately detect the* H. pylori* infection, noninvasive methods were preferred by gastroenterologists for many reasons [30–32]. The whole advantages and disadvantages of invasive and noninvasive methods are listed in detail (see Table 1). In a short sentence, the main rationale for choosing the noninvasive methods is to avoid endoscopy. Relatively high numbers of guidelines were recommending the noninvasive tests as first choice [33–35]. What should not be forgotten is that the endoscopy surgery is an unpleasant and uncomfortable approach for investigating the* H. pylori* in dyspeptic patients [36, 37]. Additionally, there are other drawbacks which limit using the invasive methods such as endoscopy; (i) patients need for 1–3 days off for this surgery, (ii) high cost for disposable forceps and other stuffs, and (iii) high risk of contamination by some viruses such as human immunodeficiency virus (HIV) and hepatitis C virus (HCV) [38, 39]. Of course, valid evidence indicating transmission of HIV and HCV among the subjects for endoscopy is not well-documented, but many patients declined this surgery just for this unpleasant probability released in media over the last years [40, 41]. Disparate distribution (patchy) of* H. pylori *in stomach is causing the bias in sampling (false negative) [1]. Indeed, taking a biopsy specimen (maximally 3-4 mm^2^) cannot guarantee the existence of* H. pylori*-colonized in stomach environment (500–1000 mm^2^ in different persons). A solution would be to increase the number of taken gastric biopsies, but for ethical limitations, gastroenterologists are highly prohibited to take 6 or more biopsies from a patient. Lastly, endoscopy is an impossible procedure for subjects such as pregnant women, children, and elderly patients [26, 42]. There are two major approaches to noninvasive tests to diagnosis the* H. pylori* infection: UBT and serological examinations [26, 43]. The main superiority of these methods is their easy applications in epidemiological studies. Furthermore, their easy application is affecting their high popularity in studies investigating the eradication rate following the antibiotic therapy [42, 44, 45]. In next paragraphs, we discuss each method separately to see their priorities and limitations in clinical settings.

## 3. Urea Breath Test

The first report of the application of UBT was about 60 years ago by Kornberg et al. investigating the intravenous injection of ^14^C urea into the cat and determining the amount of decreased ^14^C-CO_2_ in animal breath [46, 47]. Their finding was due to the colonization of* Helicobacter felis *in the cat. When ingesting ^13^C- or ^14^C-labeled exposed to the bacterial urease in* H. pylori *positive patient, hydrolyzation results in the production of CO_2_ in the stomach. Thereafter, labeled CO_2_ enters into the bloodstream and is exhaled in the lung. Consequently, the amount of trapped labeled CO_2_ will be measurable in the exhalation [48]. As mentioned above, the principle of this test is based on the intrinsic ability of* H. pylori* to break down absorbed ^13^C or ^14^C-labeled urea into CO_2_ in acidic gastric condition. If one is colonized actively with* H. pylori*, the urea is metabolized to the ammonia and labeled bicarbonate [^14^C-CO_2_]. Thereafter, labeled bicarbonate is transferred to the lung and produces labeled carbon dioxide. This product is detectable by the machine to confirm the existence of the infection. Because of high sensitivity and specificity, UBT is a very attractive method to measure the* H. pylori* active infection by microbiologists and clinicians. At least for asymptomatic subjects, the UBT is a gold standard method [49, 50]. Another preference of UBT is that the method is free of sampling errors (lack of endoscopic surgery). This superiority made it very popular for clinicians to confirm bacterial eradication, especially in asymptomatic, elderly, and pediatric subjects. Clinicians need to wait 1-2 months for performing UBT to confirm successful bacterial eradication. As noted, the false-positive result is a minor problem with UBT, and clinicians need to take care of other urease-producing organisms which may be able to change the result. Overall, the specificity and sensitivity of the UBT are mostly more than 95%. Although these high rates for both sensitivity and specificity are an advantage for this test, lack of data on antibiotic resistance and further analysis is the main limiting feature of this popular method to detect active* H. pylori* infection [51, 52]. A major consideration for this test is about its radiation. So far, decreased dosage of radiation made it a bit convenient for children but is still prohibited for pregnant women. In recent years, stool antigen test (SAT) and UBT became more acceptable diagnostic tests to detect active* H. Pylori* Infection. Lacking a universal protocol to perform UBT is an unsolved problem. Till now, only the manufacturer's experiences guided current standards in order to perform this method. Given high sensitivity (>95%) in posttreatment procedures [53], one of the disadvantages with UBT is the chance of colonization by urease-forming pathogens than* H. pylori* [54]. This probability is existing by the relatively low rate of current reports which increased our hopes to generalize application of UBT in routine and posttherapy* H. pylori* detection.

## 4. Stool Antigen Test (SAT)

Historically, serology approach was the first suggestion in order to diagnose* H. pylori* infection. Although the SAT is an accurate and precise method this accuracy is influenced by several limiting factors: upper gastrointestinal bleeding, antibiotic consumption, bowel movement, and also proton pump inhibitors (PPIs) uptake [55]. This noninvasive and almost cheap test became recommended whenever UBT was not available (Table 1). The superiority of UBT versus SAT was also found by Perri et al., while they showed that the diagnosis with UBT is undertaken with higher accuracy [56]. Sequentially, SAT was introduced following the UBT into the clinic. Polyclonal antibodies-EIA gave useful reports on the diagnostic practices but occasional inconsistent findings (mostly false-positives) forced clinicians to start application of the monoclonal antibody-based approach. An actual improvement in this technique was the higher specificity which reduced the false-positive findings [57–59]. As the constructive shift in this immunologic assay, using monoclonal antibodies provided improved sensitivity and specificity rates in comparison with UBT. There are two major preferences for SAT in comparison with UBT; less expensive chemicals and materials and also not high-technology equipment are necessary. Another advantage of this method was that patients could have stored the samples at home and send it to laboratories at a suitable time. The partial insufficient condition of preservation of the stool sample in-house (optimal temperature, shaking, and transport medium to the laboratory) beside that applied cut-off value in final measurement can determine any bias in the diagnosis of the infection. To detect* H. pylori* infection, there are two main types of SAT: based on enzyme immunoassay (EIA) and immunochromatography (ICA) [60]. In clinical practices, the ICA-based test is more convenient to run in the small clinics and hospitals since so complex procedures are involved. In 1997, this test was suggested for the first time and the found acceptable sensitivity and specificity (88% and 94%, respectively) initiate other groups to apply it over the clinical practices [61]. A very attractive advantage of this method is to measure successful eradication of the infection using a simple laboratory examination [62]. Of course, to target pediatrics, SAT using monoclonal antibodies can give better feasibility since it is of low cost and is easy to handle by regular laboratories personals. In recent years, a new generation of stool antigen tests was invented. The Testmate pylori antigen EIA and Testmate rapid pylori antigen are the major examples [63, 64]. Testmate rapid pylori antigen is an immunochromatography based approach which is located on the commercially patented test strip. This easy application is to drop of suspended stool sample into the test strip. In the case positive result, an immunologic complex with the red latex-labeled MAb 21 Ge will be produced and then it moves till it the red color line becomes visible in the test strip. Currently, there is a good agreement between published guidelines and consensuses that SAT using monoclonal antibodies is one of the best approaches in the measurement of successful eradication of the bacterium and also for primary detection of this microorganism in clinical settings [33, 43, 55, 65–67].

## 5. Serological Tests

In general, detection of specific-antibody following the exposure to the various* H. pylori* antigens can be a useful method in clinical practices. As application and logic procedure was undertaken for many other pathogenic microorganisms,* H. pylori* discovery was not far from serological diagnosis [68, 69]. To date, different bacterial components include whole cell lysate, specific outer membrane proteins, LPS, heat shock protein (HSP), catalase, and cagA protein and many of the adhesions were applied to induce specific antibodies in the host for facilitating the serological assay [60, 70–73]. Broadly defined, human immune response to the* H. pylori* is very complicated since many surface antigens are contributed. Routine* H. pylori* serologic methods can only detect specific IgG antibodies. The clinical importance of this test emerges when antibiotics and PPIs consumption are reported. Indeed, false negative results observed for other methods can have different response using serologic analysis. In addition to those drug uptakes, gastric bleeding and gastritis atrophic condition were also caused by false negative results for other methods; again, the serologic assay can be helpful for clinicians [74, 75]. The highlighted problem with the serologic approach is the weak distinguishing power of this test to discriminate active and inactive infection. Due to the different backgrounds in host genetics, it can be expected that various* H. pylori* strains induce different levels of antibodies and it may be a considerable item in explaining the reported findings [76]. Because of acceptable sensitivity and specificity rates observed in many commercial IgG-bases tests exist and have been validated in recent years [77–81]. One of the interesting aspects of serology method is following the antibiotic therapy; the long-lasting antibodies are still existing and it may cause the false-positive result [82–84]. This point should be cautiously considered by epidemiologists and gastroenterologists in their examinations in populations. In total, serologic tests are inexpensive; thus the application of these antibodies-based tests in some geographical area such as developing countries is highly acknowledged. A major consideration for the regions with a low prevalence of* H. pylori* is that suboptimal specificity can increase the false-positive results. Moreover, IgA-based measurement was also suggested but noted that the test is less trustful and reliable than IgG-based assays [85–88]. In some interesting studies, examinations of* H. pylori*-specific antibodies in other sample sources than serum were investigated [89, 90]. In brief, saliva and urine were checked but because of the lower titer of antibodies in these samples in comparison with serum, clinicians are not so eager to check this sort of samples for* H. pylori*. Taking together, the antibody-based examination cannot guarantee the accuracy of reported* H. pylori* status following the antibiotic treatment; thus further analysis is needed [91].

## 6. Invasive Methods

No need to mention that having genomic data from the clinical samples increases our knowledge of susceptibility patterns and virulence factors. Bacterial culture, Rapid urease test, PCR assay, and histology are the invasive methods applied to diagnose the* H. pylori* infection in different biologic sources. The criteria for this nomination (invasive methods) are referring to undertaking the endoscopic surgery. In patients with two complaints, the gastric endoscopy will be necessary: (i) no response to antibiotic therapy, and (ii) signs indicating problematic and symptomatic gastric conditions. Usually, clinicians take biopsies from antrum, but PPI consumption will reduce diagnostic value; thereafter, stomach body would be the next place for biopsy sampling. In next paragraphs, we discuss invasive methods used to diagnose* H. pylori* infection in clinical samples.

## 7. Histology

Histology was the first method unconsciously used to detect the* H. pylori* in clinical samples. Our main evidence is reported by Warren in his laboratory before his collaborations with Barry Marshall ended in their great discovery. However, application of Warthin–Starry silver stain by Warren eventually caused a big triumph to visualize bacterial colonization in a biopsy sample from a patient with severe gastritis [19]. Current diagnosis of* H. pylori* infection is highly influenced by the histological report [92, 93]. Since typic morphology of* H. pylori*, histopathological confirmation of this infection can be easily achieved while further histologic changes in patterns of gastritis can be helpful to characterize the digestive diseases properly. An accurate histopathological observation can give us a detailed report of possible* H. pylori* colonization (and also bacterial density), and degree of inflammation and histopathology (e.g., severe atrophic gastritis, intestinal metaplasia, and malignancy [93, 94]). The identification of the bacteria in the histopathological analysis is based on different staining including hematoxylin and eosin (A&E). In order to increase specificity in detection of the* H. pylori*, different dyes such as Gimenez, Toluidine blue, Romanowski, Genta, Warthin–Starry silver, and Giemsa can be also used [95, 96]. The specific application of Warthin–Starry silver is in the chance of coccoid forms of the* H. pylori*. Histopathology examination is basically time-consuming and relatively expensive. Thus, the requirement of trained staffs besides being a consuming process resulted in a not handy method to detect* H. pylori* infection. Both sensitivity and specificity of the histology are nearly around the 94% [97–100]. We have searched the databases (Web of Sciences, Scopus, Medline, and Google scholar) and found not that much-published reports are investigating the specificity and sensitivity of the commercially available stains. From a scientific point of view, there is no superiority for any of those tests; however, some other aspects such as rapidity, reproducibility, cost, and being less time-consuming can be favorable for some of the tests. Rotimi et al. used modified McMullen's staining as a novel approach in comparison with other staining methods but found no significant differences (*P* value > 0.05) [95]. Although we have found relatively high sensitivity for this test, patchy distribution of this bacterium in the stomach can reduce the chance of histopathological changes in the taken biopsy from antrum [101–104]. Accordingly, to maintain this high rate of sensitivity and avoid sampling errors, we need to increase the number of taken biopsies [105, 106]. Last but not least, being an experienced pathologist can increase the sensitivity of this approach. Regardless of our limitations in reduced sensitivity for histology, the typical shape of the* H. pylori* and its expectable location in the luminal surface of the cells can help pathologists for easy diagnosis of this S-shaped bacterium. There is a general agreement among the pathologists that in the case of the existence of the* H. pylori*, all staining methods are good, but modified Giemsa is the first choice because of less expensive materials and reproducible and sensitive results.

## 8. Rapid Urease Test

During the years after discovery by Marshall and Warren, it has been stated that the rapid urease test (RUT) is a one of the cheapest but rapid diagnostic tests used in detecting this infection [107]. The main biologic basis of this diagnostic test is to evaluate the presence of urease enzyme in clinical specimens shipped to the laboratories [108, 109]. Due to the historic dogma, it has hypothesized that detected gastric enzyme is a production by the human stomach to protect its epithelial cells from the acidic condition. Interestingly, following the groundbreaking introduction of* H. pylori*, it became widely accepted that the enzyme does not have a human origin [110]. Consequently, the detection of this bacterial product was used to identify this chronic infection. Interestingly, the specificity and sensitivity are durable for this test, but clinical experiences had shown that the optimal results are only achieved only if we take biopsies from both corpus and antrum. Some factors are influencing the final result of RUT: (i) bacterial urease concentration, (ii) temperature, (iii) waiting time for optimal reaction condition, and (iv) substrate concentrations.* Staphylococci* and* streptococci* are the other major urease-producers present in the gastric mucosa and may interfere with the detection of* H. pylori* based on the urease activity. There is an underestimated issue in the diagnosis of the* H. pylori* infection using the RUT. In some clinical settings mostly in developing countries, gastroenterologists ask the patients to keep the RUT test tube for 1-2 days and inform the hospitals' personals to add the data about likely positivity of the test [107, 111, 112]. In the case of oral colonization by* H. heilmannii*, the test will be positive, while it can interfere with positivity or negativity of the true* H. pylori *infection [113]. However, this reaction needs both higher load of* H. heilmannii *and longer time for positive urease reaction which mostly do not occur. Another solution to avoid this false-positive result is to check histopathological observation which is partially informative to identify* H. heilmannii* [114]. However, the necessary time to make RUT positive is quite different among numbered microorganisms; so far, a good specificity is promising news for gastroenterologists. Most of* H. pylori* contained-biopsies become red or pink within first fifty minutes after the endoscopy and placement of the biopsy in the medium [1]. The shift in the color of the medium is an indicator for the produced ammonium ion and increased pH (determined with pH indicator, e.g., phenol red). Although there are many commercial but specific mediums recommendable for detecting the urease positive organisms, some of other commonly used mediums such as Christensen medium are also useful in clinical settings [115–118]. Increasing the urea concentration (4–6 times) and reaction temperature were two potential modifications to increase the sensitivity of RUT [119]. Moreover, it has been firmly addressed that at least 10^5^ *H. pylori* isolates are required to cause a positive result in RUT (changing the color into pink). The sensitivity rates range (85–100%) made the RUT one of the highly recommendable methods in the diagnosis of the* H. pylori*. Similarly, relatively 100% specificity was another favorable item to make RUT popular among the gastroenterologists for rapid diagnosis of this bacterium in clinical settings [120].

## 9. Culture

**Figure 1:**
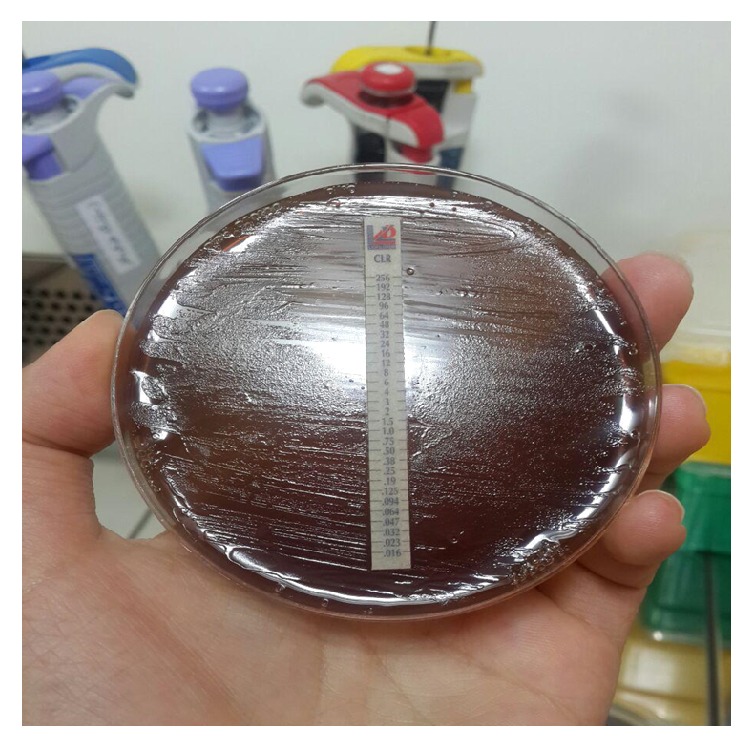
Successful isolation of* Helicobacter pylori* on Brucella agar supplemented with 5% horse blood.

Since increasing body of evidence showed the long-last colonization of the* H. pylori*, microbiologists started to culture this bacterium in several media. The main superiority of bacterial culture for* H. pylori* is the possibility of antibiotic susceptibility tests to choose proper antibiotics in the treatment of subjects and avoiding a new generation of antibiotic resistance among the symptomatic patients [121–124]. Successfulness of this culture process made this approach as the gold standard in the diagnosis of this infection. Recent international guidelines still insist on performing the bacterial culture in the case of failure in antibiotic susceptibility testing as next action. Based on Sydney classification, clinicians need at least three biopsies (two biopsies from the anterior and posterior corpus and one from antrum) necessary to accurately determine positive bacterial infection in gastritis patients [87, 93, 101, 105, 121, 125]. This kind of recommendation was another factor which initiates ones to run the routine culture for* H. pylori* in diagnostic laboratories.* H. pylori* can grow slowly on many solid media under microaerophilic condition. As a general rule,* H. pylori* needs blood or lysed blood supplements to grow optimally on agar plates (Figure 1).

Currently, Wilkins Chalgren agar, Brain heart agar, and Columbia and Brucella agars are most used base media to propagate* H. pylori* culture in routine diagnosis [26, 44, 126]. Because of the high risk of contaminating microorganisms including gram-positive microorganisms, fungi, and yeasts, using selective medium became a universal rule to have typical* H. pylori* colonies on the plates [1]. In order to increase the sensitivity and specificity of culture in the diagnosis of the* H. pylori*, we need multiple biopsies samples rather than a single antral biopsy [127]. As an additional suggestion to improve the sensitivity and specificity, the endoscopic surgery should not be performed in less than 3 months for patients who state the record of the consumption of PPI, antibiotics, and antisecretory drugs [26, 36]. Adil et al. used microcapillary, as a novel culture-based approach in detecting the* H. pylori* infection [128]. In this paper, the authors stated that microcapillary method is statistically more sensitive compared with CLO test and HE tests (*P* < 0.01). Although many attempts had been performed to optimize culture media for* H. pylori*, Adil et al. showed a novel results to standardize* H. pylori* culture using new ingredients and conditions. As one of the critical problems in the endoscopic surgery, contamination of the biopsy specimens was an annoying issue. The rationale for this consideration is about the risk of transmission of some agents including human immunodeficiency virus (HIV), hepatitis C virus (HCV), and hepatitis B virus (HBV) during the endoscopy [40]. However, using the disposable forceps in last decade decreased the chance of contamination with these infectious agents very much [129, 130]. Ecologically,* H. pylori* can survive in all sites of them stomach, but, in some cases (e.g., consumption of antisecretory drugs), the corpus will be the likely location to give us a successful culture following the endoscopy. Many medical societies are recommending that it is better to take first biopsies for culture prior to taking samples for histopathological examination. Indeed, this superiority can decrease the chance of exposure to the fixative and infectious agents influencing the chance of* H. pylori* positive culture. There is a challenging discussion among the microbiologists which is do we need to grind the biopsies before transferring the specimens into the agar plates or not? Indeed, nonhomogeneous distribution of the* H. pylori* in biopsies caused this problem. Moreover, many studies showed that there are significant differences if we use the grinded samples. The main explanation for this phenomenon is that grinding the biopsies is increasing the exposure of more* H. pylori* isolates to the favorable condition of growth, so multiple colonies will appear. In other words, isolation of* H. pylori* single colonies is feasible only if we grind the biopsies and then streak it on the plates. The importance of current recommendation is disclosed when researchers were faced with a variable pattern of genotyping from isolated DNA from a single colony in the nongrinded biopsy sample. Therefore, using the mechanical grinder and further application of single colony bacteria can increase the accuracy of the test in the diagnosis of the* H. pylori*. Meanwhile, we nicely avoid DNA-contamination by other possible agents [131]. Culturing the antral biopsy specimens is a leading item to reproduce the one of the highest sensitivities and specificities in the diagnosis of the* H. pylori*. The problems with this test are (i) likely exposure to the oxygen and temperature, expensive materials and consumables, and (iii) it is strict transport conditions. Last but not least, in close future, improving those items can help us to have better molecular techniques needing the isolated single colonies of this rouge bacterium [128].

## 10. PCR

As usual for all pathogenic microorganisms, PCR-based methods were applied to detect* H. pylori *infections in large variety of environmental and clinical samples including water, food, vegetables, human saliva, stool, gastric juice and biopsies, and dental plaques [132]. In most of those essays, housekeeping genes were used frequently to design a sensitive and specific PCR to detect* H. pylori* [133–139]. Current evidences are indicating the requirement of at least one positive test (i.e., SAT, RUT, serology, and histology) in addition to the culture positive result before we can entitle a sample as* H. pylori* positive [26]. However, having positive result from a specific PCR approach can easily replace those time-consuming and expensive tests. The main gap to achieve this point is that optimization of DNA extraction and suitable genes pattern is not validated already. For example, there are more than twenty commercial kits for DNA extraction (i.e., Yekta Tajhiz Azma, Bioneer, Sina-clon, Qiagen, and Sigma) with large variety of the DNA yields which confuse researchers in selecting the proper product. Due to the presence of* H. pylori* DNA in various biologic samples, PCR-based detection was widely used to identify this problematic infection [140–143]. Biopsy samples, saliva and gastric juice, stool, and dental plaques were frequently applied to PCR detection. The main problem with stool sample is due to the existence of billions of bacteria including chemicals, gram-positive and gram-negative, which can mostly act as inhibitor for our detection. Because of variability in different DNA extraction methods, a universal approach needs to be recommended to produce reliable results, at least in clinical settings. Another important issue is the target gene to design the primer sets. As one of global housekeeping genes, 16S rRNA was used, but many mutations have been reported which disappoint clinicians to continuous application for further analysis [144, 145]. Recently, 23S rRNA gene has been suggested due to the high sensitivity in detection of* H. pylori* in clinical samples [146, 147].

## 11. Gold Standard Methods

Currently, urease and histological analysis are considered a gold standard approach in many clinical circumstances. In other words, there is no unique method acting as a gold standard in the diagnosis of* H. pylori* infection. However, we can use UBT and SAT as highly recommended tests available among the noninvasive methods. Further shreds of evidence are necessary before we can nominate any diagnostic methods as the gold standard in various clinical circumstances of patients attributed to* H. pylori* infections.

## 12. Different Diagnostic Strategies Useful in the Detection of Bacterial Eradication

Precise identification of* H. pylori* infections among symptomatic and asymptomatic individuals was the focus of many discussions. Following increased importance of eradication of the bacteria and its positive effects ending in gastroduodenal diseases, now another question regarding the best approach to be the gold standard for detecting the* H. pylori* in treated patients is disclosed [148, 149]. Using current knowledge of antimicrobial resistance and availability of various machines, equipment, and skilled staffs [150, 151], we need more noninvasive methods to be sued for following the eradication of* H. pylori* in different targets. Thus, researches in this area will be covered by more interest.

## 13. Conclusive Remarks

Since the accurate diagnosis of* H. pylori* is idealistic view for both gastroenterologists and microbiologists, using synergistically invasive and noninvasive methods will be a future challenge in medical research topics. It is clear that recent advances in invasive and noninvasive methods for accurate diagnosis of the* H. pylori* can drastically change upcoming guidelines attributed with the management of this infection. No doubt that diagnosis of* H. pylori* infection due to its strange microniche is difficult and hard to optimize, especially for routine diagnostic. The fragility of the microorganism is another limiting factor to access necessary information on various aspects of this bacterium. Indeed, the gastroenterologists are facing a long list of various molecular and nonmolecular tests, but the problem is to optimize and design an accurate test to produce information on (i) presence of the infection and (ii) antibiotic susceptibility profile. These are the main difficulties causing complex diagnosis of* H. pylori* even if for developed countries. According to the recent European guideline, the ^13^C-UBT is nominated as the best method in the diagnosis of* H. pylori* infection. This approach shows acceptable specificity and sensitivity in clinical practice [65]. Concerning the serological examinations, the results are generalizable if local antigens were applied in the tests; otherwise, many discrepancies need further analysis. Using current knowledge of antimicrobial resistance and availability of various machines, types of equipment and skilled staffs, we need more noninvasive methods to be sued for following the eradication of* H. pylori* in different targets. Thus, researches in this area will be covered by intense interest. To achieve this point, in-depth experiments are necessary to generalize rapid and accurate molecular tests determining both bacterial identity and antibiotic resistance profile.

## Figures and Tables

**Table 1:** Overview of *H. pylori* diagnosis with invasive and noninvasive methods.

Name	Type	Reference method	Characteristics	Advantages	Disadvantages
UBT^**∗**^	Noninvasive	No	Sensitivity: >95% Specificity: >95%	(i) High specificity and sensitivity (ii) Useful to confirm H. P eradication (iii) Useful to detect gastroduodenal bleeding (iv) Relatively cheap, simple and safe (v) A gold standard only for asymptomatic patients (vi) No sampling errors, good for epidemiological studies (vii) practically useful for children ~100% sensitivity	(i) Rarely false positive results refer to urease positive organisms (ii) Radiation in the case of application of ^14^C-UBT (iii) No data about antibiotic resistance

Serology	Noninvasive	No	Sensitivity: >96% Specificity: 60–90%	(i) Has no false negative result (ii) Cheap, simple and safe (iii) Highly recommended for initial *H. pylori *screening (iv) Not affected by gastric bleeding (v) No false negative result in the case of PPI^*∗∗*^ consumption (a unique character)	(i) No data about antibiotic resistance (ii) Failure in distinguish between active and past infection (iii) No application in clinical practice and hospitals

SAT^*∗∗∗*^	Noninvasive	No	Sensitivity: >95% Specificity: >95%	(i) High specificity and sensitivity (ii) Good popularity among patients (iii) Relatively fast and simple (iv) Easy modification to produce better results (v) No need to skilled staffs	(i) No data about antibiotic resistance (ii) The false positive result in the case of PPI and antibiotics (iii) Variation in specificity and sensitivity over the different clinical circumstances

Culture	Invasive	Yes	Sensitivity: 50–95% Specificity: >95%	(i) Existing the data about antibiotic resistance (ii) High specificity but low sensitivity (the most specific method existing) (iii) The possibility of having the pure bacterium and chance of preservation for a long time	(i) Need optimal incubation conditions and highly skilled operators (ii) Fast processes after endoscopy in necessary to avoid bacterial death (iii) Risk of the false negative result in the case of PPI and antibiotic consumption (iv) Need strict condition in transport before culturing (cool temperature) (v) Time-consuming and also the most expensive method

Histology	Invasive	Yes	Sensitivity: 60–90% Specificity: >95%	(i) The gold standard for direct *H. pylori* detection (ii) Almost cheap method for using in the universal scale (iii) Simple method	(i) Contradictory results following the PPI consumption (ii) Need extra biopsy sample and facing with ethical limitations (iii) Fluorescent microscope required method (limiting wide-spread usage) (iv) The relatively high rate of false negative reports

RUT^*∗∗∗∗*^	Invasive	No	Sensitivity: 95% Specificity: 80–90%	(i) Rapid, simple and cheap method (ii) High specificity (~99%), but low sensitivity (~80%) (iii) The most handful test in a clinical setting	(i) No data about antibiotic resistance (ii) Decreased sensitivity in patients with gastric bleeding (iii) Increased false negative results in the case of antibiotics & PPI consumption and achlorhydria (iv) Not useful for screening the eradication in epidemiologic studies

PCR	Invasive	No	Sensitivity: 80% Specificity: 100%	(i) Existing data about antibiotic resistance (ii) High specificity and sensitivity (iii) Tracking the mutations involved in antibiotic resistance (iv) The possibility of virulence typing (v) Useful to detect the bacterium in environmental samples (vi) Rapid and accurate results	(i) High cost (ii) Risk of contamination (iii) Time consuming and requirement to skilled staff (low feasibility in all laboratories) (iv) Lack of data about phenotypic antibiotic susceptibility profile

^*∗*^Urea breath test; ^*∗∗*^proton pump inhibitor; ^*∗∗∗*^stool antigen test; ^*∗∗∗∗*^rapid urease test.

## References

[1] Kusters J. G., van Vliet A. H. M., Kuipers E. J. (2006). Pathogenesis of *Helicobacter pylori* infection. *Clinical Microbiology Reviews*.

[2] Hu Y., Wan J.-H., Li X.-Y., Zhu Y., Graham D. Y., Lu N.-H. (2017). Systematic review with meta-analysis: the global recurrence rate of Helicobacter pylori. *Alimentary Pharmacology & Therapeutics*.

[3] Polish L. B., Douglas J. M., Davidson A. J., Perez-Perez G. I., Blaser M. J. (1991). Characterization of risk factors for Helicobacter pylori infection among men attending a sexually transmitted disease clinic: Lack of evidence for sexual transmission. *Journal of Clinical Microbiology*.

[4] Schwarz S., Morelli G., Kusecek B. (2008). Horizontal versus familial transmission of Helicobacter pylori. *PLoS Pathogens*.

[5] Mladenova-Hristova I., Grekova O., Patel A. (2017). Zoonotic potential of Helicobacter spp.. *Journal of Microbiology, Immunology and Infection*.

[6] Elhariri M., Elhelw R., Hamza D., El-Mahallawy H. S. (2017). Serologic evidence and risk factors for Helicobacter pylori infection in animals and humans. *The Journal of Infection in Developing Countries*.

[7] Abadi A. T. B., Ierardi E., Lee Y. Y. (2015). Why do we still have Helicobacter pylori in our stomachs?. *Malaysian Journal of Medical Sciences*.

[8] Malfertheiner P., Chan F. K., McColl K. E. (2009). Peptic ulcer disease. *The Lancet*.

[9] Olbe L., Hamlet A., Dalenback J., Fandriks L. (1996). A mechanism by which Helicobacter pylori infection of the antrum contributes to the development of duodenal ulcer. *Gastroenterology*.

[10] Zhang X. Y., Mo H. Y., Huang Y. (2016). risk factor and mortality of peptic ulcer disease. *Alimentary pharmacology & therapeutics*.

[11] Smolka A. J., Schubert M. L. (2017). Helicobacter pylori-induced changes in gastric acid secretion and upper gastrointestinal disease. *Current Topics in Microbiology and Immunology*.

[12] Camilo V., Sugiyama T., Touati E. (2017). Pathogenesis of Helicobacter pylori infection. *Helicobacter*.

[13] Blaser M. J., Kobayashi K., Cover T. L., Cao P., Feurer I. D., Pérez‐pérez G. I. (1993). Helicobacter pylori infection in japanese patients with adenocarcinoma of the stomach. *International Journal of Cancer*.

[14] Abadi A. T. B. (2016). Helicobacter pylori and Gastric Cancer. *Frontiers in Medicine*.

[15] Liou J.-M., Wu M.-S., Lin J.-T. (2016). Treatment of Helicobacter pylori infection: Where are we now?. *Journal of Gastroenterology and Hepatology*.

[16] Lazar D. C., Taban S., Cornianu M., Faur A., Goldis A. (2016). New advances in targeted gastric cancer treatment. *World Journal of Gastroenterology*.

[17] Kawanaka M., Watari J., Kamiya N. (2016). Effects of Helicobacter pylori eradication on the development of metachronous gastric cancer after endoscopic treatment: Analysis of molecular alterations by a randomised controlled trial. *British Journal of Cancer*.

[18] Testerman T. L., Morris J. (2014). Beyond the stomach: an updated view of *Helicobacter pylori* pathogenesis, diagnosis, and treatment. *World Journal of Gastroenterology*.

[19] Marshall B. J., Warren J. R. (1984). Unidentified curved bacilli in the stomach of patients with gastritis and peptic ulceration. *The Lancet*.

[20] Genta R. M., Graham D. Y. (1994). Helicobacter pylori: the new bug on the (paraffin) block. *Virchows Archiv*.

[21] Abadi A. T. B. (2017). Strategies used by helicobacter pylori to establish persistent infection. *World Journal of Gastroenterology*.

[22] Fock K. M., Graham D. Y., Malfertheiner P. (2013). Helicobacter pylori research: Historical insights and future directions. *Nature Reviews Gastroenterology & Hepatology*.

[23] McMahon B. J., Bruce M. G., Koch A. (2016). The diagnosis and treatment of Helicobacter pylori infection in Arctic regions with a high prevalence of infection: Expert Commentary. *Epidemiology and Infection*.

[24] Wang Y.-K., Kuo F.-C., Liu C.-J. (2015). Diagnosis of helicobacter pylori infection: current options and developments. *World Journal of Gastroenterology*.

[25] Kim S. G., Jung H.-K., Lee H. L. (2014). Guidelines for the diagnosis and treatment of *Helicobacter pylori* infection in Korea, 2013 revised edition. *Journal of Gastroenterology and Hepatology*.

[26] Mégraud F., Lehours P. (2007). *Helicobacter pylori* detection and antimicrobial susceptibility testing. *Clinical Microbiology Reviews*.

[27] Raymond J., Thiberge J. M., Dauga C. (2016). Diagnosis of Helicobacter pylori recurrence: Relapse or reinfection? Usefulness of molecular tools. *Scandinavian Journal of Gastroenterology*.

[28] Miftahussurur M., Yamaoka Y. (2016). Diagnostic methods of Helicobacter pylori infection for epidemiological studies: critical importance of indirect test validation. *BioMed Research International*.

[29] Granstrom M., Lehours P., Bengtsson C., Mégraud F. (2008). Diagnosis of Helicobacter pylori. *Helicobacter*.

[30] Abadi A. T. B., Loffeld R. J. L. F., Constancia A. C., Wagenaar J. A., Kusters J. G. (2014). Detection of the Helicobacter pylori dupA gene is strongly affected by the PCR design. *Journal of Microbiological Methods*.

[31] Zagari R. M., Romano M., Ojetti V. (2015). Guidelines for the management of *Helicobacter pylori* infection in Italy: the III Working Group Consensus Report 2015. *Digestive and Liver Disease*.

[32] Megraud F. (2007). Helicobacter pylori and antibiotic resistance. *Gut*.

[33] Malfertheiner P., Megraud F., O'Morain C. A. (2012). Management of *Helicobacter pylori* infection—the Maastricht IV/ Florence consensus report. *Gut*.

[34] Malfertheiner P., Megraud F., O'Morain C. (2007). Current concepts in the management of Helicobacter pylori infection: the Maastricht III Consensus Report. *Gut*.

[35] Abadi A. T. B., Kusters J. G. (2016). Management of Helicobacter pylori infections. *BMC Gastroenterology*.

[36] Kiesslich R., Goetz M., Burg J. (2005). Diagnosing Helicobacter pylori in vivo by confocal laser endoscopy. *Gastroenterology*.

[37] Dinis-Ribeiro M., Areia M., De Vries A. C. (2012). Management of precancerous conditions and lesions in the stomach (MAPS): guideline from the European Society of Gastrointestinal Endoscopy (ESGE), European Helicobacter Study Group (EHSG), European Society of Pathology (ESP), and the Sociedade Portuguesa de Endoscopia Digestiva (SPED). *Endoscopy*.

[38] Kovaleva J. (2016). Infectious complications in gastrointestinal endoscopy and their prevention. *Best Practice & Research Clinical Gastroenterology*.

[39] Sakai E., Higurashi T., Ohkubo H. (2017). Investigation of Small Bowel Abnormalities in HIV-Infected Patients Using Capsule Endoscopy. *Gastroenterology Research and Practice*.

[40] Kovaleva J., Peters F. T. M., van der Mei H. C., Degener J. E. (2013). Transmission of infection by flexible gastrointestinal endoscopy and bronchoscopy.. *Clinical Microbiology Reviews*.

[41] Fischer G. E., Schaefer M. K., Labus B. J. (2010). Hepatitis C virus infections from unsafe injection practices at an endoscopy clinic in Las Vegas, Nevada, 2007-2008. *Clinical Infectious Diseases*.

[42] Ricci C., Holton J., Vaira D. (2007). Diagnosis of *Helicobacter pylori*: invasive and non-invasive tests. *Best Practice & Research Clinical Gastroenterology*.

[43] Bessède E., Arantes V., Mégraud F., Coelho L. G. (2017). Diagnosis of Helicobacter pylori infection. *Helicobacter*.

[44] Cutler A. F., Havstad S., Ma C. K., Blaser M. J., Perez-Perez G. I., Schubert T. T. (1995). Accuracy of invasive and noninvasive tests to diagnose Helicobacter pylori infection. *Gastroenterology*.

[45] Monteiro L., De Mascarel A., Sarrasqueta A. M. (2001). Diagnosis of Helicobacter pylori infection: Noninvasive methods compared to invasive methods and evaluation of two new tests. *American Journal of Gastroenterology*.

[46] Kornberg H. L., Davies R. E., Wood D. R. (1954). The activity and function of gastric urease in the cat. *Biochemical Journal*.

[47] Kornberg H. L., Davies R. E., Wood D. R. (1954). The breakdown of urea in cats not secreting gastric juice. *Biochemical Journal*.

[48] Atherton J. C., Spiller R. C. (1994). The urea breath test for Helicobacter pylori. *Gut*.

[49] Sardarian H., Fakheri H., Hosseini V., Taghvaei T., Maleki I., Mokhtare M. (2013). Comparison of hybrid and sequential therapies for helicobacter pylori eradication in iran: a prospective randomized trial. *Helicobacter*.

[50] Metanat H. A., Valizadeh S. M., Fakheri H. (2015). Comparison Between 10- and 14-Day Hybrid Regimens for Helicobacter pylori Eradication: A Randomized Clinical Trial. *Helicobacter*.

[51] Pantoflickova D., Scott D. R., Sachs G., Dorta G., Blum A. L. (2003). 13C urea breath test (UBT) in the diagnosis of Helicobacter pylori: Why does it work better with acid test meals?. *Gut*.

[52] De Francesco V., Zullo A., Perna F. (2010). Helicobacter pylori antibiotic resistance and [13C]urea breath test values. *Journal of Medical Microbiology*.

[53] Moshkowitz M., Konikoff F. M., Peled Y. (1995). High Helicobacter pylori numbers are associated with low eradication rate after triple therapy. *Gut*.

[54] Osaki T., Mabe K., Hanawa T., Kamiya S. (2008). Urease-positive bacteria in the stomach induce a false-positive reaction in a urea breath test for diagnosis of *Helicobacter pylori* infection. *Journal of Medical Microbiology*.

[55] Shimoyama T. (2013). Stool antigen tests for the management of Helicobacter pylori infection. *World Journal of Gastroenterology*.

[56] Perri F., Manes G., Neri M., Vaira D., Nardone G. (2002). Helicobacter pylori antigen stool test and 13C-urea breath test in patients after eradication treatments. *American Journal of Gastroenterology*.

[57] Gisbert J. P., De La Morena F., Abraira V. (2006). Accuracy of monoclonal stool antigen test for the diagnosis of H. pylori infection: a systematic review and meta-analysis. *American Journal of Gastroenterology*.

[58] Simoons-Smit I. M., Appelmelk B. J., Verboom T. (1996). Typing of Helicobacter pylori with monoclonal antibodies against Lewis antigens in lipopolysaccharide. *Journal of Clinical Microbiology*.

[59] Koletzko S., Konstantopoulos N., Bosman D. (2003). Evaluation of a novel monoclonal enzyme immunoassay for detection of Helicobacter pylori antigen in stool from children. *Gut*.

[60] Konstantopoulos N., Rüssmann H., Tasch C. (2001). Evaluation of the Helicobacter pylori stool antigen test (HpSA) for detection of Helicobacter pylori infection in children. *American Journal of Gastroenterology*.

[61] Makristathis A., Pasching E., Schutze K., Wimmer M., Rotter M. L., Hirschl A. M. (1998). Detection of *Helicobacter pylori* in stool specimens by PCR and antigen enzyme immunoassay. *Journal of Clinical Microbiology*.

[62] Vaira D., Malfertheiner P., Mégraud F. (1999). Diagnosis of Helicobacter pylori infection with a new non-invasive antigen-based assay. *The Lancet*.

[63] Shimoyama T., Sawaya M., Ishiguro A., Hanabata N., Yoshimura T., Fukuda S. (2011). Applicability of a rapid stool antigen test, using monoclonal antibody to catalase, for the management of *Helicobacter pylori* infection. *Journal of Gastroenterology*.

[64] Calvet X., Lázaro M.-J. R., Lehours P., Mégraud F. (2013). Diagnosis and epidemiology of *Helicobacter pylori* infection. *Helicobacter*.

[65] Malfertheiner P., Megraud F., O’Morain C. A., Gisbert J. P., Kuipers E. J., Axon A. T. (2017). Management of Helicobacter pylori infection—the Maastricht V/Florence Consensus Report. *Gut*.

[66] Asaka M., Kato M., Takahashi S.-I. (2010). Guidelines for the management of *Helicobacter pylori* infection in Japan: 2009 revised edition. *Helicobacter*.

[67] Chey W. D., Wong B. C. Y. (2007). Practice Parameters Committee of the American College of G. American College of Gastroenterology guideline on the management of Helicobacter pylori infection. *The American Journal of Gastroenterology*.

[68] Jones D. M., Lessells A. M., Eldridge J. (1984). Campylobacter like organisms on the gastric mucosa: Culture, histological, and serological studies. *Journal of Clinical Pathology*.

[69] Rathbone B. J., Wyatt J. I., Worsley B. W. (1986). Systemic and local antibody responses to gastric Campylobacter pyloridis in non-ulcer dyspepsia. *Gut*.

[70] Mitchell H. M., Hazell S. L., Li Y., Hu P. (1996). Serological response to specific Helicobacter pylori antigens: Antibody against CagA antigen is not predictive of gastric cancer in a developing country. *American Journal of Gastroenterology*.

[71] Camorlinga-Ponce M., Torres J., Perez-Perez G. (1998). Validation of a serologic test for the diagnosis of *Helicobacter pylori* infection and the immune response to urease and CagA in children. *American Journal of Gastroenterology*.

[72] Torres J., Leal-Herrera Y., Perez-Perez G. (1998). A community-based seroepidemiologic study of *Helicobacter pylori* infection in Mexico. *The Journal of Infectious Diseases*.

[73] Khanna B., Cutler A., Israel N. R. (1998). Use caution with serologic testing for Helicobacter pylori infection in children. *The Journal of Infectious Diseases*.

[74] Shin C. M., Kim N., Lee H. S. (2009). Validation of diagnostic tests for *Helicobacter pylori* with regard to grade of atrophic gastritis and/or intestinal metaplasia. *Helicobacter*.

[75] Oksanen A., Veijola L., Sipponen P., Schauman K.-O., Rautelin H. (1998). Evaluation of Pyloriset Screen, a rapid whole-blood diagnostic test for Helicobacter pylori infection. *Journal of Clinical Microbiology*.

[76] Vaira D., Vakil N. (2001). Blood, urine, stool, breath, money, and Helicobacter pylori. *Gut*.

[77] Lee S.-Y., Moon H.-W., Hur M., Yun Y.-M. (2015). Validation of western Helicobacter pylori IgG antibody assays in Korean adults. *Journal of Medical Microbiology*.

[78] Yee Y. K., Yip K. T., Que T. L. (2002). Efficacy of enzyme immunoassay for the detection of *Helicobacter pylori* antigens in frozen stool specimens: local validation. *Alimentary Pharmacology & Therapeutics*.

[79] Breslin N. P., Lee J. M., Buckley M. J., Balbirnie E., Rice D., O'Morain C. A. (2000). Validation of serological tests for Helicobacter pylori infection in an irish population. *Irish Journal of Medical Science*.

[80] Moayyedi P., Carter A. M., Catto A., Heppell R. M., Grant P. J., Axon A. T. R. (1997). Validation of a rapid whole blood test for diagnosing *Helicobacter pylori* infection. *British Medical Journal*.

[81] Blecker U., Lanciers S., Hauser B. (1993). Validation of a new serologic test for the detection of Helicobacter pylori. *Acta Gastro-Enterologica Belgica*.

[82] Lahner E., Bordi C., Di Giulio E. (2002). Role of Helicobacter pylori serology in atrophic body gastritis after eradication treatment. *Alimentary Pharmacology & Therapeutics*.

[83] Pérez-Pérez G. I., Cutler A. F., Blaser M. J. (1997). Value of serology as a noninvasive method for evaluating the efficacy of treatment of Helicobacter pylori infection. *Clinical Infectious Diseases*.

[84] Cutler A., Schubert A., Schubert T. (1993). Role of Helicobacter pylori serology in evaluating treatment success. *Digestive Diseases and Sciences*.

[85] Pandya H. B., Patel J. S., Agravat H. H., Singh N. K. R. (2014). Non-invasive diagnosis of helicobacter pylori: Evaluation of two enzyme immunoassays, testing serum IgG and IgA response in the Anand district of central Gujarat, India. *Journal of Clinical and Diagnostic Research*.

[86] Li S., Lu A.-P., Zhang L., Li Y.-D. (2003). Anti-Helicobacter pylori immunoglobulin G (IgG) and IgA antibody responses and the value of clinical presentations in diagnosis of H. pylori infection in patients with precancerous lesions. *World Journal of Gastroenterology*.

[87] Veenendaal R. A., Götz J. M., Schroijen V. (1995). Diagnosis of Helicobacter pylori infection by specific gastric mucosal IgA and IgG pylori antibodies. *Journal of Clinical Pathology*.

[88] Granberg C., Mansikka A., Lehtonen O.-P. (1993). Diagnosis of Helicobacter pylori infection by using Pyloriset EIA-G and EIA-A for detection of serum immunoglobulin G (IgG) and IgA antibodies. *Journal of Clinical Microbiology*.

[89] Berloco P., Cavallini A., Di Leo A., Russo F. (2001). Saliva samples not a reliable tool for diagnosis of Helicobacter pylori infection. *European Journal of Clinical Microbiology & Infectious Diseases*.

[90] Christie J. M. L., McNulty C. A. M., Shepherd N. A., Valori R. M. (1996). Is saliva serology useful for the diagnosis of Helicobacter pylori?. *Gut*.

[91] Korean H. (1998). Diagnosis and treatment of Helicobacter pylori infection in Korea. *The Korean Journal of Gastroenterology*.

[92] Dooley C. P., Cohen H., Fitzgibbons P. L. (1989). Prevalence of helicobacter pylori infection and histologic gastritis in asymptomatic persons. *The New England Journal of Medicine*.

[93] Price A. B. (1991). The Sydney System: histological division. *Journal of Gastroenterology and Hepatology*.

[94] Desmet V. J., Gerber M., Hoofnagle J. H., Manns M., Scheuer P. J. (1994). Classification of chronic hepatitis: diagnosis, grading and staging. *Hepatology*.

[95] Rotimi O., Cairns A., Gray S., Moayyedi P., Dixon M. F. (2000). Histological identification of Helicobacter pylori: Comparison of staining methods. *Journal of Clinical Pathology*.

[96] Laine L., Lewin D. N., Naritoku W., Cohen H. (1997). Prospective comparison of H&E, Giemsa, and Genta stains for the diagnosis of *Helicobacter pylori*. *Gastrointestinal Endoscopy*.

[97] Shrestha R., Batajoo K., Koirala K., Shiv Raj K. (2014). Helicobacter pylori infection among patients with upper gastrointestinal symptoms: prevalence and relation to endoscopy diagnosis and histopathology. *Journal of Family Medicine and Primary Care*.

[98] Khedmat H., Karami A., Safiri Z. (2012). Helicobacter pylori genotypes can predict gastric tissue histopathology: A longitudinal study of Iranian patients. *Journal of Infection and Public Health*.

[99] Tham K. T., Peek R. M., Atherton J. C. (2001). Helicobacter pylori genotypes, host factors, and gastric mucosal histopathology in peptic ulcer disease. *Human Pathology*.

[100] Nogueira C., Figueiredo C., Carneiro F. (2001). Helicobacter pylori genotypes may determine gastric histopathology. *The American Journal of Pathology*.

[101] Gottrand F., Cullu F., Turck D. (1997). Normal gastric histology in Helicobacter pylori-infected children. *Journal of Pediatric Gastroenterology and Nutrition*.

[102] Grove D. I., Koutsouridis G., Cummins A. G. (1998). Comparison of culture, histopathology and urease testing for the diagnosis of *Helicobacter pylori* gastritis and susceptibility to amoxycillin, clarithromycin, metronidazole and tetracycline. *Pathology*.

[103] Resende L. M., Queiroz D. M., Barbosa A. J., Mendes E. N., Rocha G. A., Coelho L. G. (1993). Histology of the mucosa of gastric antrum and body before and after eradication of Helicobacter pylori. *Brazilian journal of medical and biological research = Revista brasileira de pesquisas medicas e biologicas*.

[104] Ito S., Kohli Y., Kato T. (1993). Histology of Helicobacter pylori (Hp) infection: gastric local immunity to Hp. *Nippon rinsho. Japanese journal of clinical medicine *.

[105] Nowak J. A. (1995). Limitation of histology for detecting Helicobacter pylori. *Gastrointestinal Endoscopy*.

[106] Mégraud F. (2009). Advantages and Disadvantages of Current Diagnostic Tests for the Detection of. *Scandinavian Journal of Gastroenterology*.

[107] Marshall B. J., Warren J. R., Francis G. J., Lang ton S. R., Goodwin C. S., Blincow E. D. (1987). Rapid Urease Test in the Management of Campylobacter pyloridis‐Associated Gastritis. *American Journal of Gastroenterology*.

[108] Raj P., Thompson J. F., Pan D. H. (2017). Helicobacter pylori serology testing is a useful diagnostic screening tool for symptomatic inner city children. *Acta Paediatrica*.

[109] Kashani N., Talebi Bezmin Abadi A. (2017). Reliability of rapid urease test for screening gastric cancer in high-risk populations. *Scandinavian Journal of Gastroenterology*.

[110] Mobley H. L. T., Cortesia M. J., Rosenthal L. E., Jones B. D. (1988). Characterization of urease from Campylobacter pylori. *Journal of Clinical Microbiology*.

[111] Lam S. K., Talley N. J. (1998). Report of the 1997 Asia Pacific Consensus Conference on the management of Helicobacter pylori infection. *Journal of Gastroenterology and Hepatology*.

[112] Howden C. W., Hunt R. H. (1998). Guidelines for the management of Helicobacter pylori infection. *American Journal of Gastroenterology*.

[113] Okiyama Y., Matsuzawa K., Hidaka E., Sano K., Akamatsu T., Ota H. (2005). Helicobacter heilmannii infection: Clinical, endoscopic and histopathological features in Japanese patients. *Pathology International*.

[114] Dieterich C., Bouzourène H., Blum A. L., Corthésy-Theulaz I. E. (1999). Urease-based mucosal immunization against Helicobacter heilmannii infection induces corpus atrophy in mice. *Infection and Immunity*.

[115] Shepard M. C., Lunceford C. D. (1976). Differential agar medium (A7) for identification of Ureaplasma urealyticum (human T mycoplasmas) in primary cultures of clinical material. *Journal of Clinical Microbiology*.

[116] Testerman T. L., McGee D. J., Mobley H. L. T. (2001). Helicobacter pylori growth and urease detection in the chemically defined medium Ham's F-12 nutrient mixture. *Journal of Clinical Microbiology*.

[117] Mobley H. L. T., Hu L.-T., Foxall P. A. (1991). Helicobacter pylori urease: Properties and role in pathogenesis. *Scandinavian Journal of Gastroenterology*.

[118] Midolo P., Marshall B. J. (2000). Accurate diagnosis of *Helicobacter pylori*. Urease tests. *Gastroenterology Clinics of North America*.

[119] Boyanova L., Stancheva I., Todorov D. (1996). Comparison of three urease tests for detection of Helicobacter pylori in gastric biopsy specimens. *European Journal of Gastroenterology & Hepatology*.

[120] Abadi A. T. B., Taghvaei T., Wolfram L. (2011). Inefficiency of rapid urease test for confirmation of Helicobacter pylori. *Saudi Journal of Gastroenterology*.

[121] Perez-Trallero E., Montes M., Alcorta M., Zubillaga P., Telleria E. (1995). Non-endoscopic method to obtain Helicobacter pylori for culture. *The Lancet*.

[122] Xia H. X., Keane C. T., O'Morain C. A. (1994). Culture of Helicobacter pylori under aerobic conditions on solid media. *European Journal of Clinical Microbiology & Infectious Diseases*.

[123] Ho B., Vijayakumari S. (1993). A simple and efficient continuous culture system for Helicobacter pylori.. *Microbios*.

[124] Assous M., Zone A., Watine J., Paul G., Guerre J. (1991). Frozen biopsy specimens and culture rates of Helicobacter pylori. *The Lancet*.

[125] Best L. M., Haldane D. J. M., Bezanson G. S., Veldhuyzen van Zanten S. J. O. (1997). *Helicobacter pylori*: primary susceptibility to clarithromycin in vitro in Nova Scotia. *Canadian Journal of Gastroenterology & Hepatology*.

[126] Ohara S. (2009). Diagnostic methods for H. pylori infection. *Nippon rinsho. Japanese journal of clinical medicine *.

[127] Genta R. M., Graham D. Y. (1994). Comparison of biopsy sites for the histopathologic diagnosis of Helicobacter pylori: a topographic study of H. pylori density and distribution. *Gastrointestinal Endoscopy*.

[128] Allahverdiyev A. M., Bagirova M., Caliskan R. (2015). Isolation and diagnosis of Helicobacter pylori by a new method: Microcapillary culture. *World Journal of Gastroenterology*.

[129] Rutala W. A., Weber D. J. (2004). Reprocessing endoscopes: United States perspective. *Journal of Hospital Infection*.

[130] Sakurai Y., Nakatsu M., Sato Y., Sato K. (2003). Endoscope contamination from HBV- and HCV-positive patients and evaluation of a cleaning/disinfecting method using strongly acidic electrolyzed water. *Digestive Endoscopy*.

[131] Israel D. A., Salama N., Krishna U. (2001). Helicobacter pylori genetic diversity within the gastric niche of a single human host. *Proceedings of the National Acadamy of Sciences of the United States of America*.

[132] Zhang W.-H., Wang Y., Guo H.-F., Bai Y.-J., Yan X.-J. (2005). Detection of antibody against Helicobacter pylori UreB by fluorescence polarized immunoassay using single epitope synthetic peptide as antigen. *Xi bao yu fen zi mian yi xue za zhi = Chinese journal of cellular and molecular immunology*.

[133] Taghvaei T., Talebi Bezmin Abadi A., Ghasemzadeh A., Naderi B. K., Mohabbati Mobarez A. (2012). Prevalence of horB gene among the Helicobacter pylori strains isolated from dyspeptic patients: First report from Iran. *Internal and Emergency Medicine*.

[134] Bharath T. S., Reddy M. S., Dhanapal R., Kumar N. G. R., Raju P. V. N., Saraswathi T. R. (2014). Molecular detection and corelation of Helicobacter pylori in dental plaque and gastric biopsies of dyspeptic patients. *Journal of Oral and Maxillofacial Pathology*.

[135] Dadashzadeh K., Milani M., Rahmati M., Akbarzadeh A. (2014). Real-time PCR detection of 16S rRNA novel mutations associated with Helicobacter pylori Tetracycline resistance in Iran. *Asian Pacific Journal of Cancer Prevention*.

[136] Gold B. D., Gilger M. A., Czinn S. J. (2014). New Diagnostic Strategies for Detection of Helicobacter pylori Infection in Pediatric Patients. *Gastroenterology & hepatology*.

[137] Holman C. B., Bachoon D. S., Otero E., Ramsubhag A. (2014). Detection of Helicobacter pylori in the coastal waters of Georgia, Puerto Rico and Trinidad. *Marine Pollution Bulletin*.

[138] Kaymakçı M., Aydın M., Yazıcı S., Sağır O., Gür O. E. R., Sayan M. (2014). Detection of Helicobacter pylori in adenoid tissue by real-time polymerase chain reaction. *Kulak burun boğaz ihtisas dergisi : KBB = Journal of ear, nose, and throat*.

[139] Trespalacios A. A., Rimbara E., Otero W., Reddy R., Graham D. Y. (2015). Improved allele-specific PCR assays for detection of clarithromycin and fluoroquinolone resistant of Helicobacter pylori in gastric biopsies: Identification of N87I mutation in GyrA. *Diagnostic microbiology and infectious disease*.

[140] Redondo J. J., Keller P. M., Zbinden R., Wagner K. (2018). A novel RT-PCR for the detection of Helicobacter pylori and identification of clarithromycin resistance mediated by mutations in the 23S rRNA gene. *DIAGNOSTIC MICROBIOLOGY AND INFECTIOUS DISEASE*.

[141] Khadangi F., Yassi M., Kerachian M. A. (2017). Review: Diagnostic accuracy of PCR-based detection tests for Helicobacter Pylori in stool samples. *Helicobacter*.

[142] Talarico S., Safaeian M., Gonzalez P. (2016). Quantitative Detection and Genotyping of Helicobacter pylori from Stool using Droplet Digital PCR Reveals Variation in Bacterial Loads that Correlates with cagA Virulence Gene Carriage. *Helicobacter*.

[143] Ahmad S., Ahmad F., Rahman F. U. (2016). PCR based detection of phase variable genes in Pakistani based clinical Helicobacter pylori strains. *Jundishapur Journal of Microbiology*.

[144] Lu J.-J., Perng C.-L., Shyu R.-Y., Chen C.-H., Lou Q., Chong S. K. (1999). Comparison of Five PCR Methods for Detection ofHelicobacter pylori DNA in Gastric Tissues. *Journal of clinical microbiology*.

[145] Hoshina S., Kahn S. M., Jian W. (1990). Direct detection and amplification of Helicobacter pylori ribosomal 16S gene segments from gastric endoscopic biopsies. *Diagnostic microbiology and infectious disease*.

[146] Van Doorn L.-J., Glupczynski Y., Kusters J. G. (2001). Accurate prediction of macrolide resistance in Helicobacter pylori by a PCR line probe assay for detection of mutations in the 23S rRNA gene: Multicenter validation study. *Antimicrobial Agents and Chemotherapy*.

[147] Ho S.-A., Hoyle J. A., Lewis F. A. (1991). Direct polymerase chain reaction test for detection of *Helicobacter pylori* in humans and animals. *Journal of Clinical Microbiology*.

[148] Atkinson N. S. S., Braden B. (2016). Helicobacter Pylori Infection: Diagnostic Strategies in Primary Diagnosis and After Therapy. *Digestive Diseases and Sciences*.

[149] Lario S., Ramírez-Lázaro M. J., Montserrat A. (2016). Diagnostic accuracy of three monoclonal stool tests in a large series of untreated Helicobacter pylori infected patients. *Clinical Biochemistry*.

[150] Hirschl A. M., Makristathis A. (2007). Methods to detect Helicobacter pylori: From culture to molecular biology. *Helicobacter*.

[151] Lage A. P., Godfroid E., Fauconnier A. (1995). Diagnosis of Helicobacter pylori infection by PCR: Comparison with other invasive techniques and detection of cagA gene in gastric biopsy specimens. *Journal of Clinical Microbiology*.

